# A data driven approach for soft tissue biomarker identification linked to Chiari-like malformation and syringomyelia

**DOI:** 10.3389/fvets.2024.1492259

**Published:** 2025-01-22

**Authors:** Jake Cumber, Emma Scales-Theobald, Clare Rusbridge, Kevin Wells

**Affiliations:** ^1^Centre for Vision Speech and Signal Processing (CVSSP), University of Surrey, Guildford, United Kingdom; ^2^Canine Chiari Group, School of Veterinary Medicine, Faculty of Health and Medical Sciences, Guildford, United Kingdom; ^3^Fitzpatrick Referrals Orthopaedics and Neurology, Godalming, United Kingdom; ^4^Wear Referrals Veterinary Specialist and Emergency Hospital, Stockton-on-Tees, United Kingdom

**Keywords:** brachycephaly, canine, image registration, machine learning, morphologies, MRI

## Abstract

Canine Chiari-like malformation (CM) is a neuroanatomical condition associated with conformational change of the cranium, craniocervical junction and neuroparenchyma, resulting in pain (Chiari associated pain or CM-P) and the development of syringomyelia (SM). The associated neuro-disability in affected individuals compromises quality of life. CM is characterized by overcrowding of the brain and cervical spinal cord and is predisposed by skull-base shortening and miniaturization with brachycephalic toy dogs overwhelmingly represented. Magnetic resonance imaging (MRI) is conventionally used for diagnosis; however, CM is complex and ubiquitous in some dog breeds so that diagnosis of CM-P relies on a combination of clinical signs, MRI, and elimination of other causes of pain. This research aimed to identify cranial and spinal pathologies and neural morphologies linked to CM-P and SM in dogs using MRI scans and machine learning with the aim of identifying novel data driven biomarkers which could confirm CM-P and identify dogs at risk of developing SM. The methodology identified four regions of interest as having robust discrimination for CM-P, with 89% sensitivity and 76% specificity. A set of morphological features linked to CM-P were identified. Four regions of interest were also identified as having robust discrimination for SM, with 84% sensitivity and 80% specificity. Overall, these findings shed light on the distinct morphologies related to CM-P and SM, offering the potential for more accurate and objective diagnoses in affected dogs using MRI. These results contribute to the further understanding of the complex pathologies associated with CM and SM in brachycephalic toy pure and mixed breed dogs and support the potential utility of data-driven techniques for advancing our knowledge of these debilitating conditions.

## 1 Introduction

Canine Chiari-like malformation (CM) is a complex developmental condition of the skull and craniocervical vertebrae characterized by a conformational change with overcrowding of the brain and spinal cord, particularly at the craniospinal junction (1–3). The bony abnormalities associated with CM are a combination of brachycephaly (skull base shortening), craniofacial hypoplasia, caudal (posterior) fossa insufficiency and craniocervical junction abnormalities. A degree of CM is ubiquitous or extremely common in predisposed toy breed dogs (both purebred and crossbred). Research by Cerda-Gonzalez et al. (4) found 92% of MRIs from clinically normal Cavalier King Charles spaniels (CKCS) dogs showed at least one craniocervical morphologic abnormality. Some dogs are free of clinical signs but dogs with more extreme brachycephaly can have clinical signs of pain typically associated with Valsalva mimics (manifest as vocalization on rapid postural changes including being picked up, jumping, and shifting position when recumbent) and signs suggesting back of head, ear or facial pain (scratching or rubbing these areas), reduced activity levels, spinal pain and behavioral changes. This collection of signs is referred to as CM-associated pain (CM-P) (5). The signs of CM-P can lead to a drastic reduction in the affected canine's quality of life (6).

Morphological changes linked to CM can also serve as a risk factor for the disruption to the flow of cerebrospinal fluid (CSF), a consequence of this disruption is fluid cavitation in the spinal cord: known as syringomyelia (SM). These cavities (singular: syrinx, plural: syringes) are comprised of a fluid, similar to CSF, that if large can result in myelopathy and a central spinal cord syndrome. This is characterized by pain including allodynia, fictive scratching (a maladaptive scratch reflex associated with syrinx involvement of the mid cervical superficial dorsal horn), thoracic limb and spinal weakness and cervico-torticollis (7). Brachycephalic toy dogs that are predisposed to CM will therefore be at greater risk of having both CM-P and SM, and as such are often studied in tandem (8). For example, one study in Griffon Bruxellois dogs found 61.7% of those with CM also suffered from SM (9). Additionally, CM is a heritable condition (10), and SM has a moderately high estimate of heritability (11), both of which can become progressively worse with each generation (12). Therefore, it is important to detect these conditions in dogs to suitably inform breeding choices for breeders and ideally prevent these conditions from being passed on.

The diagnosis of CM-P and SM currently requires the use of magnetic resonance imaging (MRI) (13). Syringomyelia is straightforward to identify and can be measured objectively as the maximum transverse width of the syrinx (13). This has been previously correlated with likelihood of clinical signs (7). However, diagnosis of CM-P relies on a combination of clinical signs, MRI, and elimination of other causes of pain and is more subjective (13). Many research teams have explored the morphologies linked to both CM-P and SM using tools from statistical analysis (2), morphometric measurements from MRI and CT (3, 14–16), and machine learning (1, 17). Morphometric mapping, such as that used in (3, 14), works to identify key areas of the brain associated with the conditions and be used as a diagnostic tool, however, this is a lengthy process. Machine learning can automate this process and has the potential to detect subtle common patterns within the data that human operators may not perceive. For example, work by Knowler et al. (1) highlighted the importance of assessing MRI scans of the whole head, rather than solely the hindbrain, for diagnosis. Spiteri et al. (17) attempted to derive a data-driven method to analyze local neuromorphologies adjacent to the hindbrain linked to CM-P and grades of SM using techniques from machine learning; this identified key areas diagnostically relevant to the conditions and was able to successfully discriminate between conditions. The aim of this latest study is to further extend and enhance the approach originally proposed by Spiteri et al. (17) to search for novel discriminative regions linked to CM-P and/or SM across the entire sagittal image using a purely data-driven approach.

## 2 Materials and methods

Midsagittal T2 weighted MRI slices of one-hundred and twenty Cavalier King Charles spaniels (CKCS) dogs were provided from one veterinary practice, and included 63 males and 57 females, ranging from 1.3 to 9.7 years. Information on the dogs included is available in Table 1. The data was collected using a 1.5 T MRI scanner (Siemens Symphony Mastro Class, Enlargen, Germany) with the default slice thickness obtained being 3.2 mm with a pixel resolution of 0.7 mm by 0.7 mm in the sagittal plane and was T2-weighted. The MRI series captured the full head in all subjects, terminating at the C4 vertebrae (*n* = 59) or up to the C6 vertebrae (*n* = 61). The data was anonymized with demographic data pertaining to the subjects' age and gender being retained. An ECVN diplomat and international expert on CM-P and SM graded each subject for CM-P and SM status and assigned them to one of three groups based on their physical exam, medical history, and the full MRI imaging of the entire neuroaxis according to a syringomyelia protocol (13):

- (CM-N): Considered clinically normal for the breed and at least 3 years old. MRI may show signs of mild CM, but no history or signs of CM-P and no syrinx present (*n* = 34).
- (CM-P): CM identified on MRI. Clinical history and exam suggested CM pain with other diagnoses ruled out. Central canal diameter < 1 mm. For this study syringomyelia is defined as a central canal dilatation of 1 mm or more (*n* = 51).
- (SM-S): CM identified on MRI. Clinical history and exam suggested clinical signs associated with SM with or without CM-P. Syrinx maximum transverse diameter > 4 mm detected (*n* = 35).

**Table 1:** Demographics for all participating dogs (*n* = 120), separated into study categories: CM-N (*n* = 34), CM-P (*n* = 51), and SM-S (*n* = 35).

**CM/SM status**	**Age**			**Sex**	
	≤ **3 years**	**3–5 years**	≥**5 years**	**Male**	**Female**
CM-N	7	11	16	26	8
CM-P	13	17	21	21	30
SM-S	11	8	16	16	19

The in-between phenotype with a small diameter syrinx (1–4 mm maximum transverse diameter) (so called SM-mild or SM-M) was excluded because these dogs are typically presented with clinical signs of pain and are less likely to have signs directly related to SM. This study was submitted for ethics and governance review to the University of Surrey NASPA Ethics Committee and granted a favorable ethical opinion (NASPA-2018-005).

**Figure 1:**
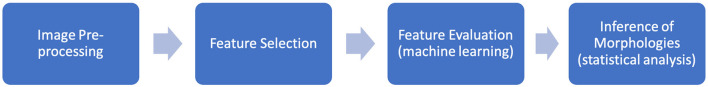
Schematic diagram highlighting the key steps within the research pipeline.

The methodology for this research can be partitioned into four key steps: image preprocessing, feature selection, feature evaluation (machine learning) and inference of morphologies, summarized in Figure 1. This methodological pipeline allowed for an assumption-free (i.e., purely data-driven) assessment of morphologies linked to disorder and exploration into the dominant morphological trends linked to disorder. All experimental processes were carried out using MATLAB ver. 9.13.0 R2022b (18).

The image pre-processing step is based around selecting a reference CM-N average dog. This will be used for warping (using image registration methods) MRI images of all other dogs in the dataset to this individual and then using machine learning to reveal and highlight subtle morphological patterns associated with CM-P and SM-S within the data. The feature selection step was then implemented, whereby a map of pixel displacements created by the image warping/registration process for each dog were explored to identify local candidate regions that may be predictive of CM-P/SM-S. The feature evaluation step used machine learning to identify the most highly predictive candidate regions. These regions were further refined to improve predictive performance. This enabled the morphologies predictive of CM-P/SM-S to be isolated. Each step is detailed below.

### 2.1 Image preprocessing

Our approach is based on mapping mid-sagittal MRI images to a suitable reference subject, and from this, using machine learning to infer where morphological abnormalities are located which may be associated with CM-P or SM-S. The use of a reference subject was favored to preserve definition of all anatomical features, especially those of a smaller size and/or variable position based on age/weight or other factors which would be during any averaging process.

**Figure 2:**
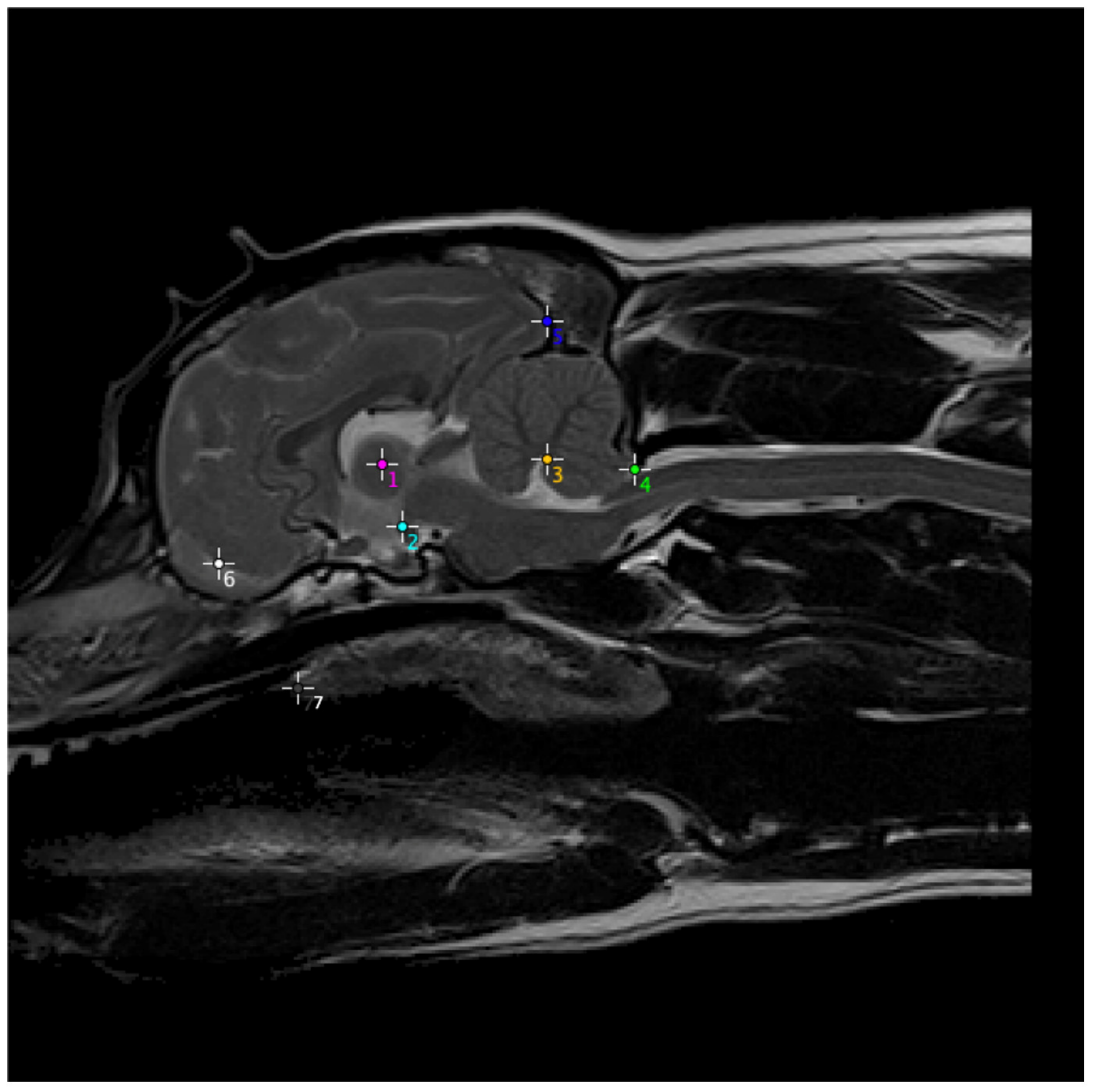
Anatomical structures used to generate a set of morphometric measurements, on one example subject, which were then computed for every CM-N subject and analyzed using principal components analysis (PCA), to identify the optimum reference dog. (1) Center of intra-thalamic adhesion; (2) mamillary body of hypothalamus (directly above caudal border of sella turcica and at rostral and ventral edge of midbrain); (3) apex of 4th Ventricle; (4) tip of caudal cerebellar vermis (at junction between parenchyma and CSF); (5) occipital pole; (6) center of CSF space between rostral frontal lobe and olfactory bulb; (7) junction between hard and soft palate. The most rostral point of soft palate.

A suitable reference dog was selected, deemed to be a CKCS that was clinically unaffected by CM-P and SM (i.e., CM-N group), as well as exhibiting no other known head morphological diseases. To identify the optimum reference dog, a set of key anatomical landmarks were manually annotated for all 34 CM-N subjects in the dataset and analyzed using principal components analysis (PCA). The annotation located the center of mass of seven key anatomical structures chosen for repeatability of identification, as shown in Figure 2. These anatomical landmarks were selected based on ease of identification and repeatability across different subjects.

The reference dog's age and weight were subsequently compared to the average age and weight data from the CM-N subjects to ensure it was also representative of an average of the group. The average age and weight (and standard deviation) of dogs in the CM-N class were 4.9 years (± 1.8 years) and 11.4 kg (± 3.6 kg), respectively, with the reference dog having an age of 5.1 years (53% percentile) and a weight of 11.9 kg (54% percentile).

The selection of the reference dog allowed for subsequent image registration of all subjects in the study (CM-N, CM-P and SM-S) to the reference dog, from which a deformation map for each subject was produced. This was achieved by first undertaking a rigid-body registration consisting of rotation and translation operations to ensure all subjects images were in the same approximate initial alignment. The rigid-registration steps ensured the center of each image corresponded to the center of intra-thalamic adhesion (point 1 in Figure 2) and that all subjects are facing the same direction as the reference subject. Once all subjects were rigidly registered to the reference dog, non-rigid Demons registration was performed (19).

**Figure 3:**
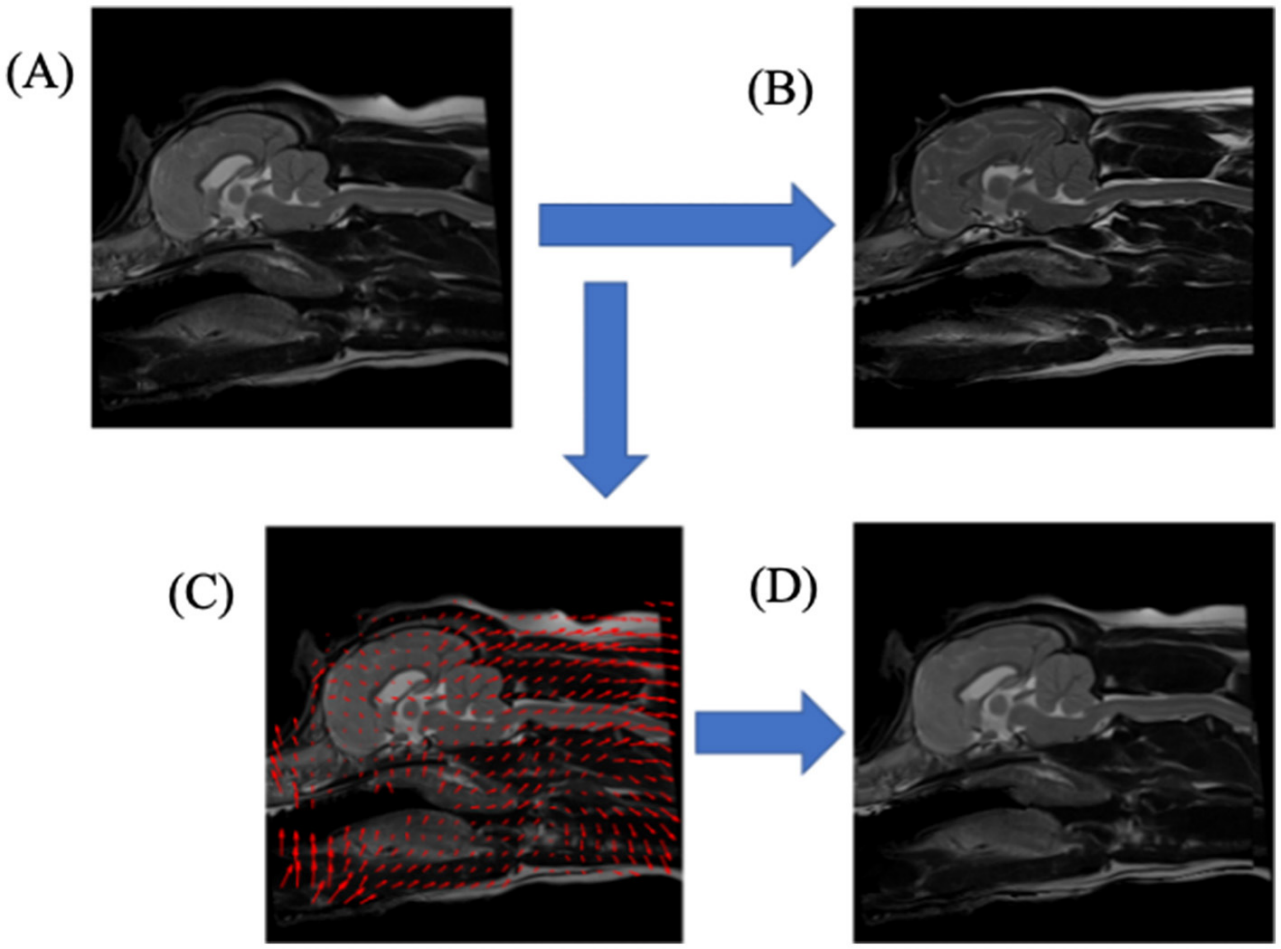
A schematic diagram illustrating the non-rigid registration process, Demons deformation, aligning one T2-weighted mid-sagittal MRI slice to another. **(A)** upper-left image, shows query dog prior to registration; **(B)** upper-right image, shows the reference dog image to which the query dog **(A)** would be registered; **(C)** lower-left image shows a down-sampled illustration of the deformation image mapping the query dog **(A)** to reference dog **(B)**; **(D)** lower-right image, shows the result of the transformations from **(C)** applied to query dog **(A)**.

Demons registration is a non-rigid technique which applies non-linear transformations to images to “squeeze and squash” a target image to best align it with the reference image; an example of demons deformation can be found in Figure 3. This produced an aligned image of a query image mapped to the reference image, a deformation map representing the individual pixel displacements that map the query image to the reference image, and a reverse aligned reference image, which mapped the reference image to the query image.

### 2.2 Feature selection

Spiteri et al. (17), observed that the severity (i.e., magnitude) of pixel displacements seen in the deformation maps were more significant for predictive purposes than simple (*x*, y) displacements or the angles of the pixel displacements. Therefore, this approach was adopted in this latest work.

**Figure 4:**
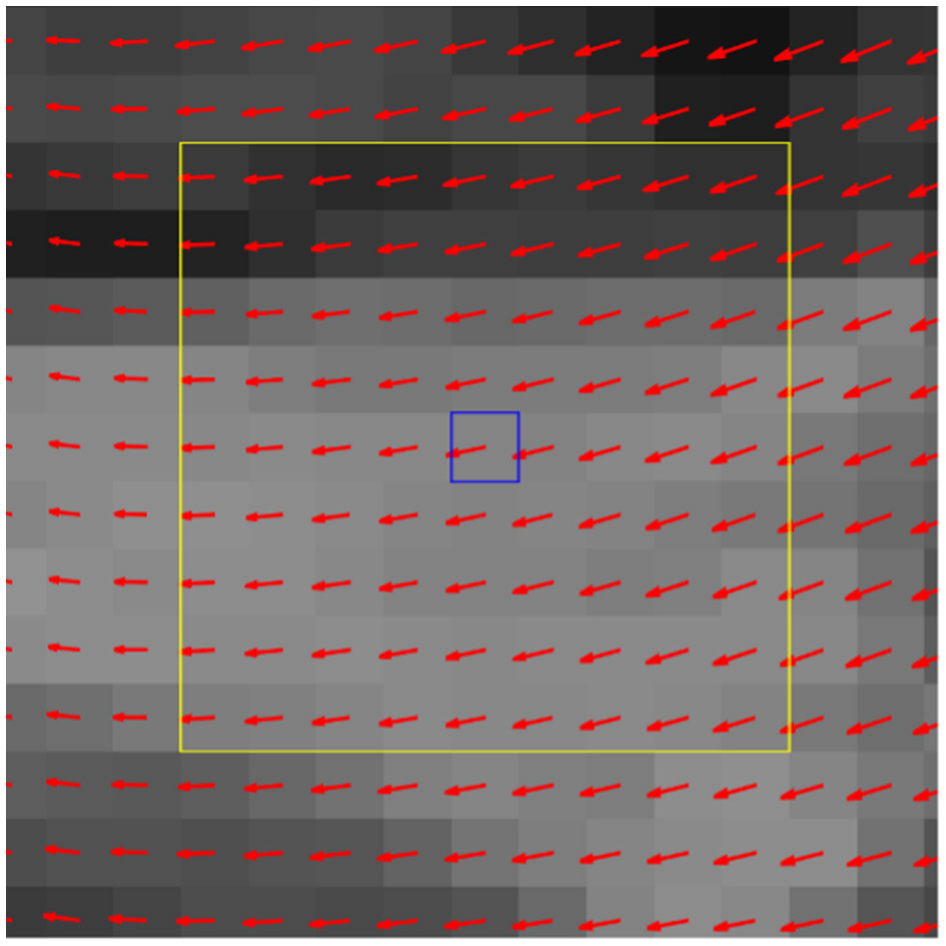
Exemplar deformation field with center pixel (blue box) and its locale (yellow box). In the feature evaluation step, the deformation data from corresponding locales are collected from all query dogs and serve as the 81 selected features from the 9 × 9 locale for a machine learning (ML) classifier.

We then systematically examined every pixel location and its local neighborhood as a potential candidate biomarker for neuromorphological abnormality. We refer to these as a seed point centered on a local pixel neighborhood (locale); previous pilot investigations demonstrated that a 9 × 9 region, as shown in Figure 4, yielded the best predictive performance. These local displacement maps were then used to build a local set of machine-learned (rather than human prescribed) features that could be used to predict presence of CM-P/SM-S. This process was then repeated across the entire image, stepping one pixel at a time to build up thousands of local models for prediction.

### 2.3 Feature evaluation

To evaluate the aforementioned local displacement maps for their potential to predict CM-P and SM-S, a machine learning (ML) algorithm was implemented. We selected a support vector machine (SVM) (20) as the preferred approach. Experiments to detect morphologies linked to CM and SM were carried out separately: the CM experiment treats CM-N as controls and CM-P as disease subjects, and the SM experiment treats CM-N as controls and SM-S as disease subjects.

Each local displacement maps, identified by its central pixel location or seed point was evaluated numerically using receiver operating characteristic (ROC) curve, from which the area under the curve (AUC) score was calculated and assigned to each seed point. A seed point with an AUC score approaching 1.0 (100% accuracy) represents perfect predictive performance, a score approaching 0.5 (50% accuracy) would be deemed analogous to random guessing.

For machine learning, the datasets are to be partitioned into training and testing sets, according to a ratio of approximately 80/20 where the training data is used by the SVM to learn, and the test data is reserved to monitor how well the SVM has performed. Initially the SVM will use all training data at once (per locale) which is followed by cross-validation to gain an understanding of how well the predictive models performed when trained on different subsets of training data. The entire training dataset was grouped into eight “folds”. Each fold was allowed to train on 7/8 of the training data, and the remaining 1/8 data was used as an interim validation set. This was repeated across all combinations of train and validation folds to examine how predictive value varies across different subjects within the dataset, and so indicate how well such an approach might work when deployed in a clinical setting. The reserved test data is only then introduced to the model and its performance noted.

**Figure 5:**
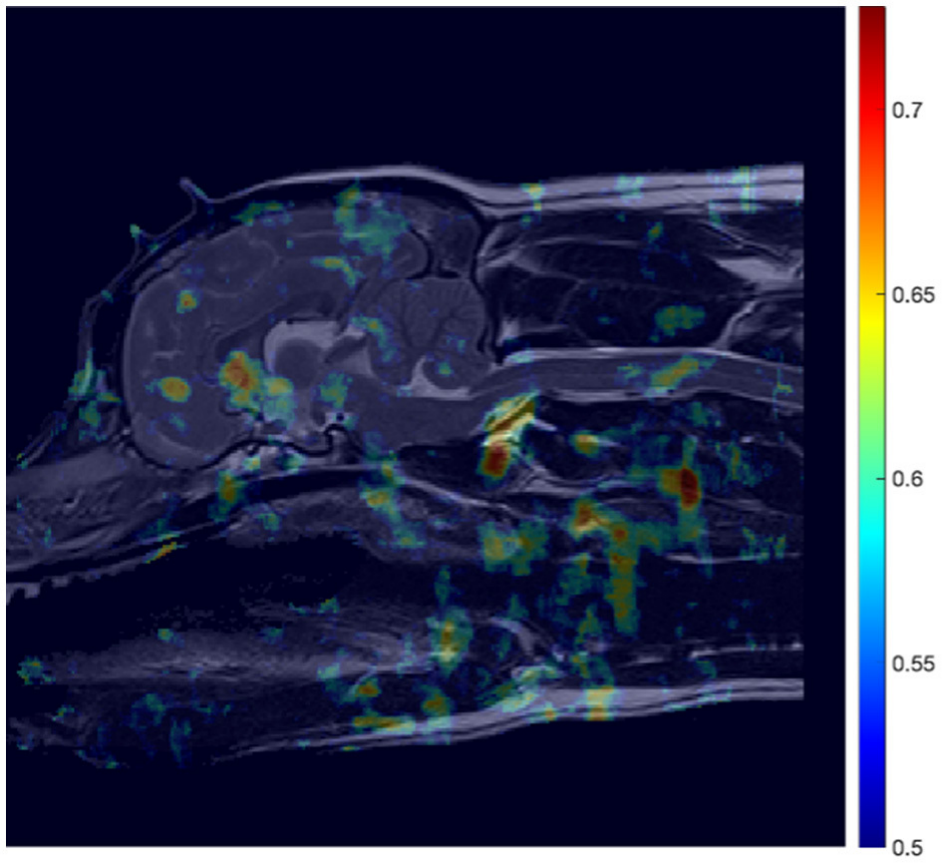
AUC heatmap overlayed onto reference image (Figure 3B) showing potential regions of interest (ROIs) in the detection of CM-P related morphologies. The AUC scores attributed to individual pixels represent those averaged during a cross-validation process, to assess performance of the ML model at predicting CM-P presence. The resultant heatmap is thresholded to remove noise by only showing distinction between regions whose AUC scores exceed 0.5 up to the maximum recorded score of 0.73.

The average performance across all folds, in terms of AUC was then visually expressed by mapping individual pixel performance across the entire reference image, as exemplified in Figure 5 for the CM-P study. Rather than rely on a single region as the sole arbiter of CM-P or SM-S presence, we then sought to aggregate the best performing regions in an optimized manner to enhance overall performance of the ML model. To do so, the map of AUC performance was thresholded at an AUC score of 0.62. This was deliberately set relatively low so that potentially small, highly predictive, regions tempered by adjacent regions with relatively poor predictive value would not be inadvertently removed. Each remaining region was then repeatedly dilated to include a new one-pixel wide boundary, and subsequently tested for AUC performance until the AUC score of the dilated region plateaued. At this point each pixel adjoining the dilated boundary was then individually tested to examine whether its inclusion increased or decreased overall AUC performance. Increases in performance meant the pixel was appended to the region, and decreases resulted in exclusion. This process was continued until maximum performance for the region was attained. This produced a set of expanded regions with consistently enhanced AUC performance compared to those in the initial thresholding step.

In order to combine these regions in a way that improved upon individual region performance, we then embarked on an exhaustive search of all possible combinations of these modified regions, from each one considered individually up to all regions jointly. Each combination was evaluated using the AUC score following training. The resulting combination of region(s) from this brute force search yielding the highest possible AUC score during training were chosen as the final regions of interest.

### 2.4 Inference of morphologies

The final stage is to understand the local deformation information found in the identified ROI's, to understand the morphologies that are linked to disease/disorder. In the case for each disorder, an application of principal components analysis (PCA) was employed, to identify the most salient deformations within the ROI's. This was visualized using a vector line plot overlayed onto the reference subject and allows for the comparison between control and disease subjects, thus allowing for the direct comparison of natural morphologies with disorder-indicative morphologies.

## 3 Results

### 3.1 CM-P analysis

**Figure 6:**
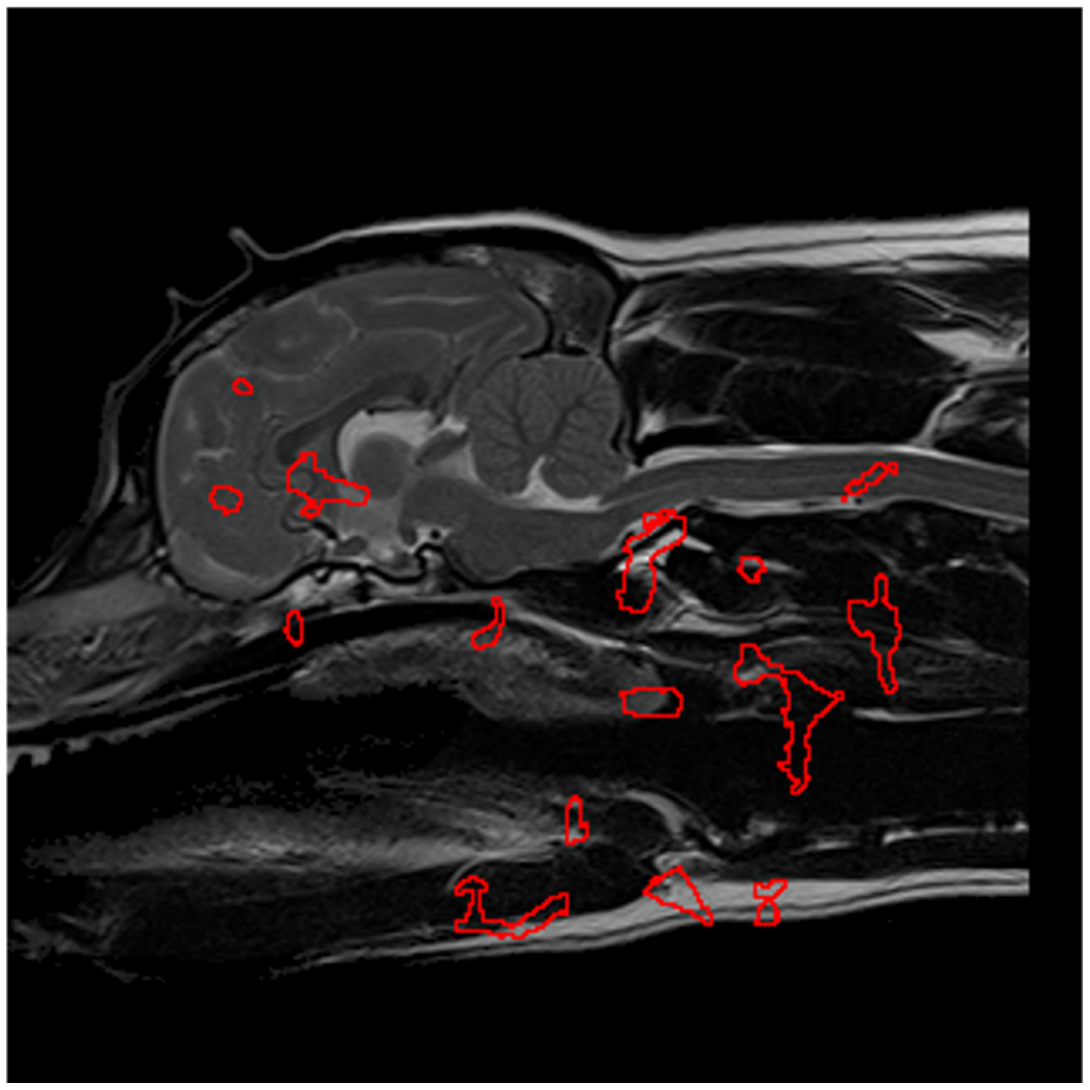
The most significant regions, extracted from Figure 5, in the detection of CM-P morphologies, are enveloped in red and serve as the designated regions of interest in further experimentation.

**Figure 7:**
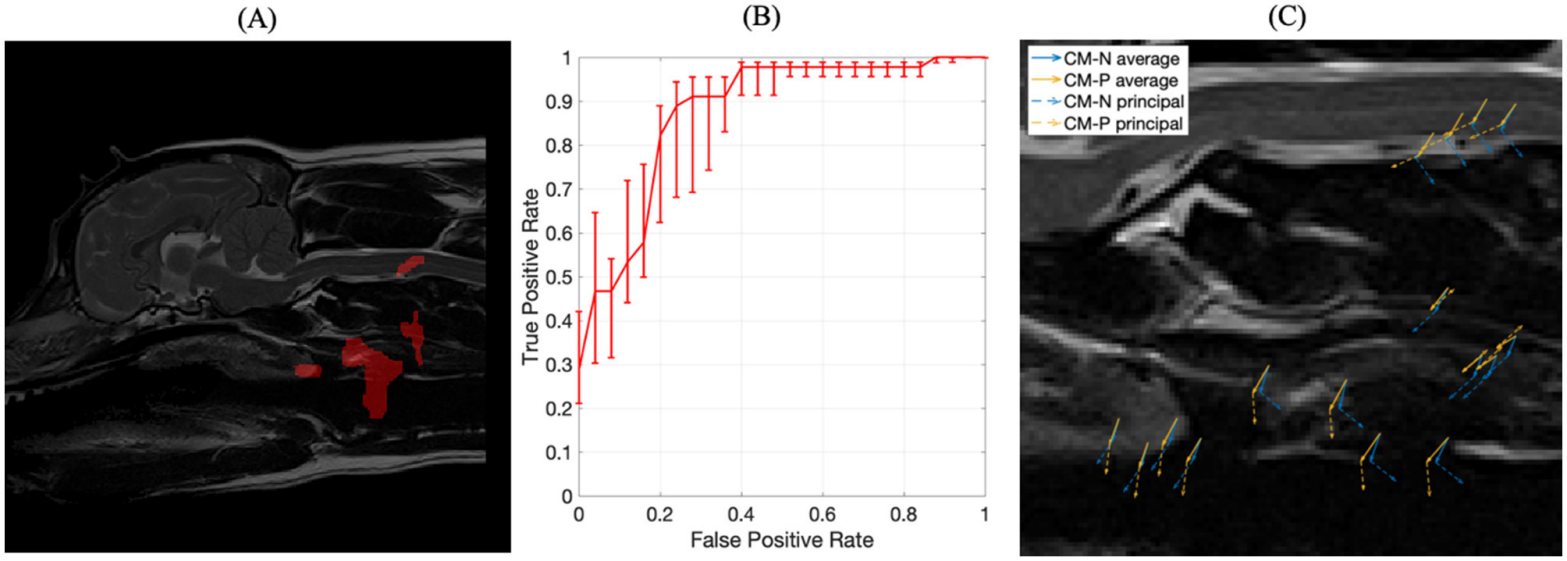
**(A)** The four optimized ROIs with highest combined predictive performance for CM-P presence superimposed on the reference dog; **(B)** corresponding ROC curve for the combined ROIs indicated in **(A)**; **(C)** visual representation of exemplar locations selected from each of the regions shown in **(A)** and the associated deformation directions for CM-N subjects (blue) and deformations linked to CM-P (orange), with solid lines indicating average displacements and dashed lines indicating the prominent (principal) direction to which most deformations can be measured against.

In the case of CM-P, the performance of the individual seed points, evaluated in the feature evaluation stage, were visually expressed as a heatmap as shown in Figure 5. The thresholding of AUC scores exceeding 0.62 resulted in the fifteen solitary regions found in Figure 6. The optimization of the ROI boundaries to maximize AUC score added an average of 6.1 pixels per region and excluded an average of 4.2 pixels per region; the resultant ROI's were visually indistinguishable from those shown in Figure 6; the median improvement in AUC scores during this process was 0.019. The brute force search for region combinations found that four ROI's were the most highly predictive of CM-P presence: these can be seen in Figure 7A. Surprisingly, all four predictive regions were outside the brain and three were outside nervous system, these ROI's being (1) the caudal end of the soft palate (AUC = 0.70), (2) larynx and the soft tissue directly dorsal the larynx (AUC = 0.73), and (3) the fascia containing the carotid artery caudal to the bifurcation and ventral to caudal C2 (AUC = 0.81). Additionally, there was a region (4) at the level of the C3 spinal cord segment (spinal cord associated with the caudal third of the C2 vertebral body) (AUC = 0.64).

The machine trained on the union of these four regions' morphological data yielded an AUC score of 0.87, an improvement between 0.06 and 0.23 compared to individual regions. The corresponding receiver operating characteristic (ROC) curve, shown in Figure 7B, emits an operating point yielding 89% sensitivity and 76% specificity. This shows the machine has an 89% accuracy at producing a true positive diagnosis.

A PCA analysis was used to better understand the morphological changes seen in the four key selected regions that were predictive of CM-P, with results shown in Figure 7C. The first principal component from this analysis, shown in orange for CM-P presence against CM-P absence (CM-N) shown in blue, explained 91% of the variance within the dataset. The visual summary of these CM-P morphologies is plotted in Figure 7C, highlighting the average displacements and prominent direction most deformations can be measured against. When exploring the dominant morphological behaviors in the ROI's, we are able to understand how these can vary between CM-N and CM-P dogs. Morphologies in region (1) of the soft palate show ventral displacements with separation of 31 degrees between CM-N and CM-P; region (2) at the larynx shows CM-N and CM-P exhibiting caudally directed and ventrally directed morphologies, respectively. Region (3) at the facia containing the carotid artery shows directly opposing morphologies between CM-N and CM-P subjects, directed ventrally and dorsally, respectively. Region (4) situation within the C2/C3 spinal cord shows CM-N related morphologies point ventrally/caudally, with CM-P subjects directed ventrally/rostrally.

### 3.2 SM-S analysis

**Figure 8:**
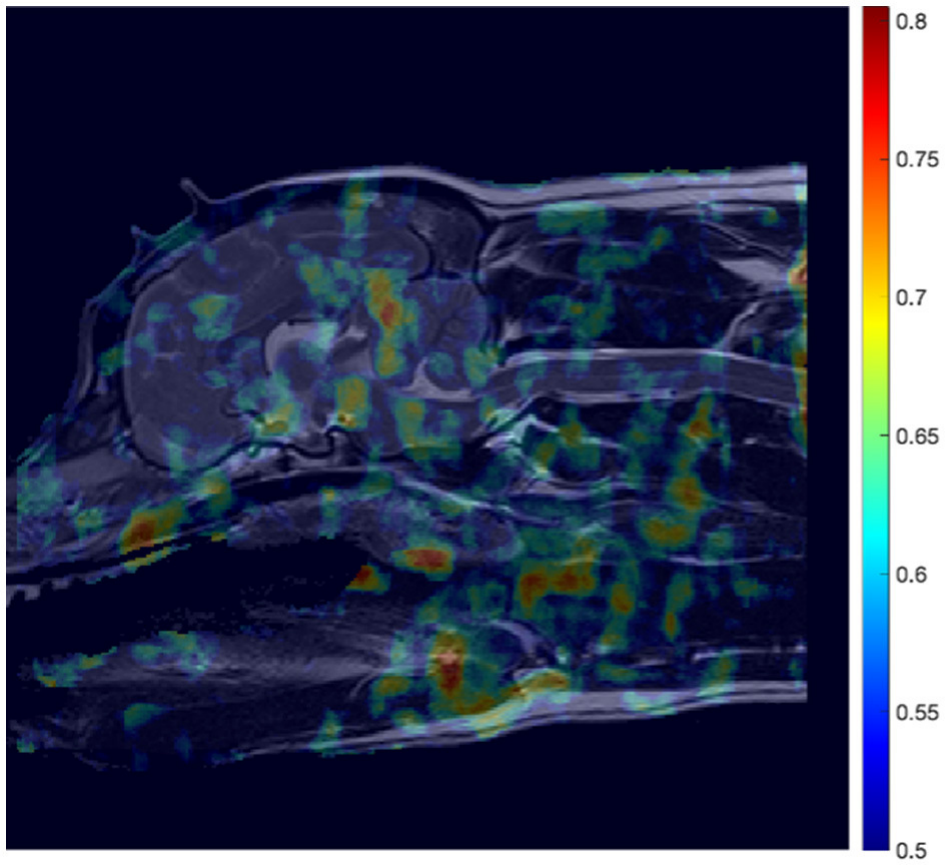
Mosaic-style heatmap overlayed onto reference image (Figure 3B) showing potential regions of interest (ROIs) in the detection of SM-S related morphologies. The attributed scores are a result of area under the curve (AUC) scores, averaged during a cross-validation process, and thresholded to only show distinction between regions whose AUC scores exceed 0.5, and up to the maximum recorded score of 0.805.

**Figure 9:**
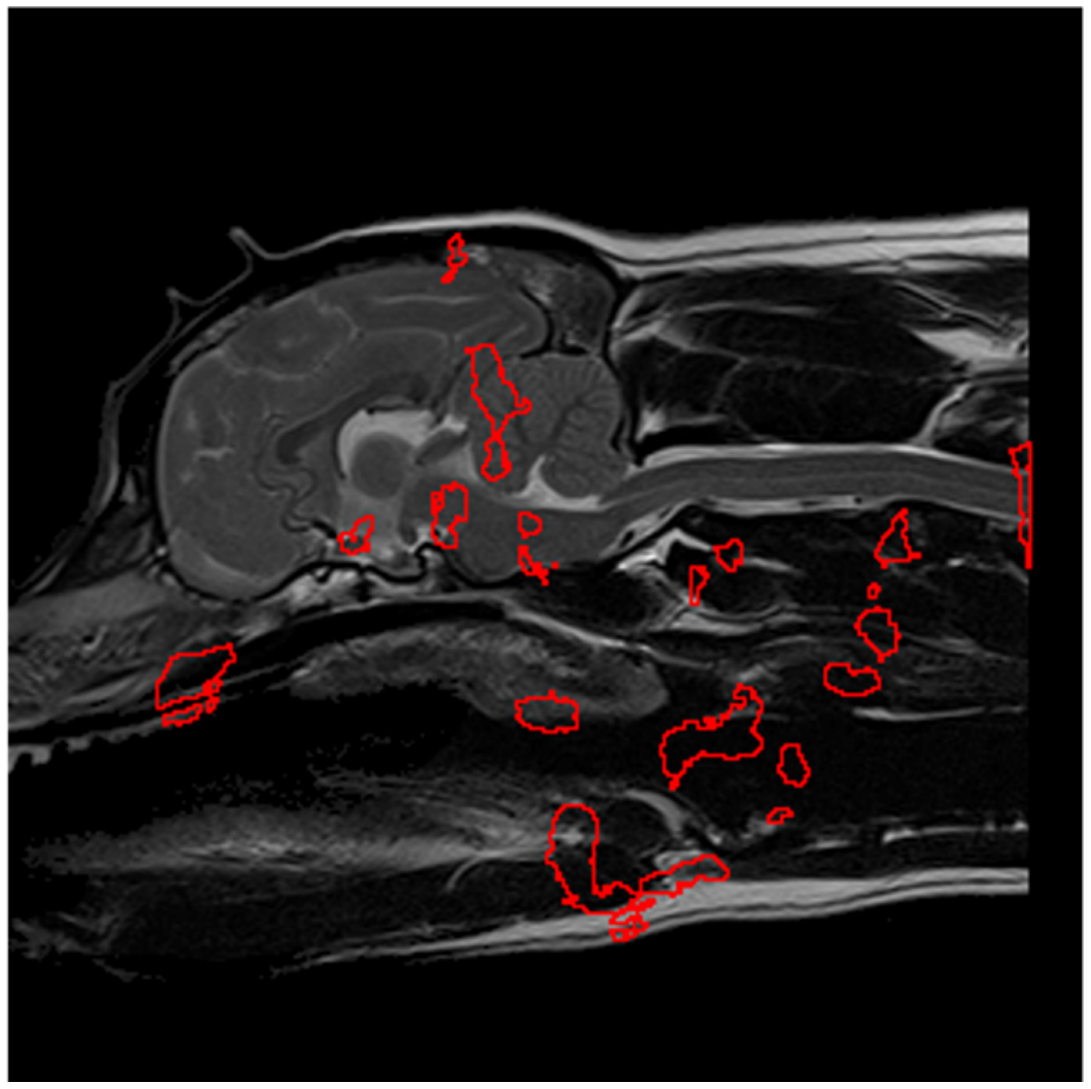
The most significant regions for predicting SM presence, extracted from Figure 8 and shown in red, which serve as preliminary regions of interest pending further experimentation.

The aforementioned methodology was also applied to develop a ML model dedicated to predicting SM presence. This resulted in an AUC heatmap (see Figure 8) revealing far more regions attaining AUC scores exceeding 0.62 than in the CM-P case. Regions with a threshold AUC score exceeding 0.62 were isolated and are shown in Figure 9. The aforementioned methodology was also applied to develop a ML model dedicated to predicting SM presence. This resulted in an AUC heatmap (see Figure 8) revealing far more regions attaining AUC scores exceeding 0.62 than in the CM-P case. Regions with a threshold AUC score exceeding 0.62 were isolated and are shown in Figure 9. The boundaries of the twenty-one regions were optimized, revealing that several ROIs could be merged by including the adjacent pixels shared between them. This process resulted in fourteen strongly predictive candidate ROIs associated with SM-S; the median improvement in AUC scores as a result of the optimization was 0.021.

**Figure 10:**
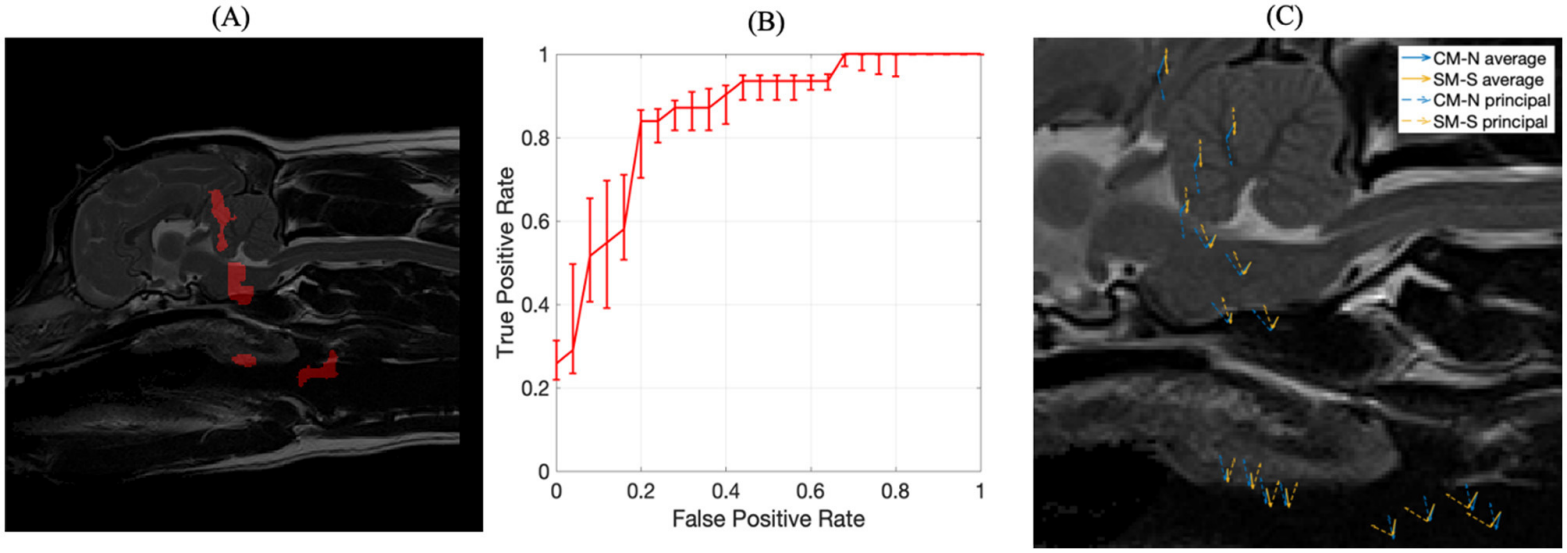
The inference of ROIs for SM-S. **(A)** left image, the combination of regions resulting in the strongest discriminating ability for SM-S morphologies, obtained with an exhaustive permutative search; **(B)** center image, the corresponding ROC curve for the ROI morphologies indicated in **(A)**; **(C)** visual representation of exemplar locations selected from each of the regions shown in **(A)** and the associated deformation directions for CM-N subjects (blue) and deformations linked to SM-S (orange), with solid lines indicating average displacements and dashed lines indicating the prominent (principal) direction to which most deformations can be measured against.

All combinations of regions were then examined for combined predictive performance in terms of AUC score. The best performing combination of ROIs for predicting morphologies associated with SM-S were determined to be the four ROI's with the strongest discriminating ability shown in Figure 10A. Two of these regions were located within the brain being (1) the rostral cerebellum and tentorium cerebelli region (AUC = 0.77) and (2) brain stem immediately ventral to the fourth ventricle (AUC = 0.78). There were two regions outside the nervous system being (3) rostral to the soft palate ROI found in the CM-P study (AUC = 0.74) and (4) the epiglottal region of the larynx (AUC = 0.71), as summarized in Figure 10A.

The corresponding ROC curve for this combination of ROIs is shown in Figure 10B, which yields an AUC score of 0.85 (an improvement of between 0.07 and 0.14) and a sensitivity of 84% and specificity of 80%. This indicates the ML model demonstrates 84% accuracy at producing a true positive diagnosis. We were able to bootstrap the test data with the CM-P dataset, allowing for fifty-one dogs to be tested; it was found that the model correctly classified 76% of CM-P dogs as not being SM-S, consistent with the existing specificity noted during initial testing. The principal morphologies attributed to these ROIs linked to SM-S, as shown in Figure 10C and explain 94% of all inscribed morphologies.

The analysis of the morphological behaviors found in these four regions also showed key differences between CM-N and SM-S subjects. Region (1) at the tentorium cerebelli shows a clear dorsal morphology indicative of SM-S directly opposed by a ventral morphology linked to CM-N; with similarly dorsal morphologies linked to SM-S in region (2) at the brain stem. Region (3) located at the soft palate shows a 40-degree separation between CM-N and SM-S morphologies and region (4) in the larynx shows a 60-degree separation between CM-N and SM-S subjects' morphologies.

## 4 Discussion

An objective and clinically relevant grading of CM and the diagnosis of CM-associated pain (CM-P) has been the subject of many research studies with still no clear consensus among the community. A deeper understanding of the morphological changes to the skull, cervical vertebrae and underlying nervous tissue and how they result in CM-P and syringomyelia (SM) will lead to greater ability for subjects to be more easily diagnosed, properly treated earlier in life, and screened to assess the likelihood of disease transmission through offspring. In this work we have sought to identify data-driven regions that exhibit soft tissue deformations that are predictive for CM-P and SM when compared to CM-N individuals.

The consistency of a single neurologist assessing the MRI's obtained from a single center using a standardized protocol allowed for the minimization of inter-operator variance. Therefore, a machine learning algorithm could be trained independent of previous veterinary knowledge on the morphological information found in registering subjects to a reference dog. This approach was subsequently refined to optimize the identified regions of interest (ROI) and to concurrently verify the optimum set of ROIs linked to disease. The statistical analysis of the morphological differences between CM-N and CM-P/SM-S subjects also allowed for the potential to learn far more relating to the pathophysiology of both CM-P and SM-S giving greater clinical relevance. Overall, up to 89% accuracy in the CM-P analysis and 80% accuracy in the SM study during this testing phase shows there is a high confidence in the identified ROIs, and their morphologies, for both diseases. This represents state-of-the-art predictive performance or these two conditions.

### 4.1 CM-P morphologies and clinical relevance

It was surprising to observe that all major predictive regions were outside the nervous system, and this shows the value of a machine learning approach that is not led by human bias or hypothesis. However, it is difficult to explain the clinical signs of CM-P with these morphologies. It is most likely a para-phenomenon and that the conformational changes in the skull are also resulting in conformational changes in the upper airway and associated soft tissue. This has been described in humans with the comparable condition Chiari type 1 malformation (21) and Chiari malformation associated with complex craniosynostosis (22). An alternative explanation may be that the confirmation change to the airway may be affecting intrathoracic pressure and sleep which in turn may affect cerebrospinal fluid movement and clinical signs associated with Valsalva maneuver mimics such as rapid postural changes including being picked up, jumping, shifting position when recumbent, coughing and defecation. Likewise changes in conformation of the great blood vessels may affect blood and lymphatic drainage from the head (23).

**Figure 11:**
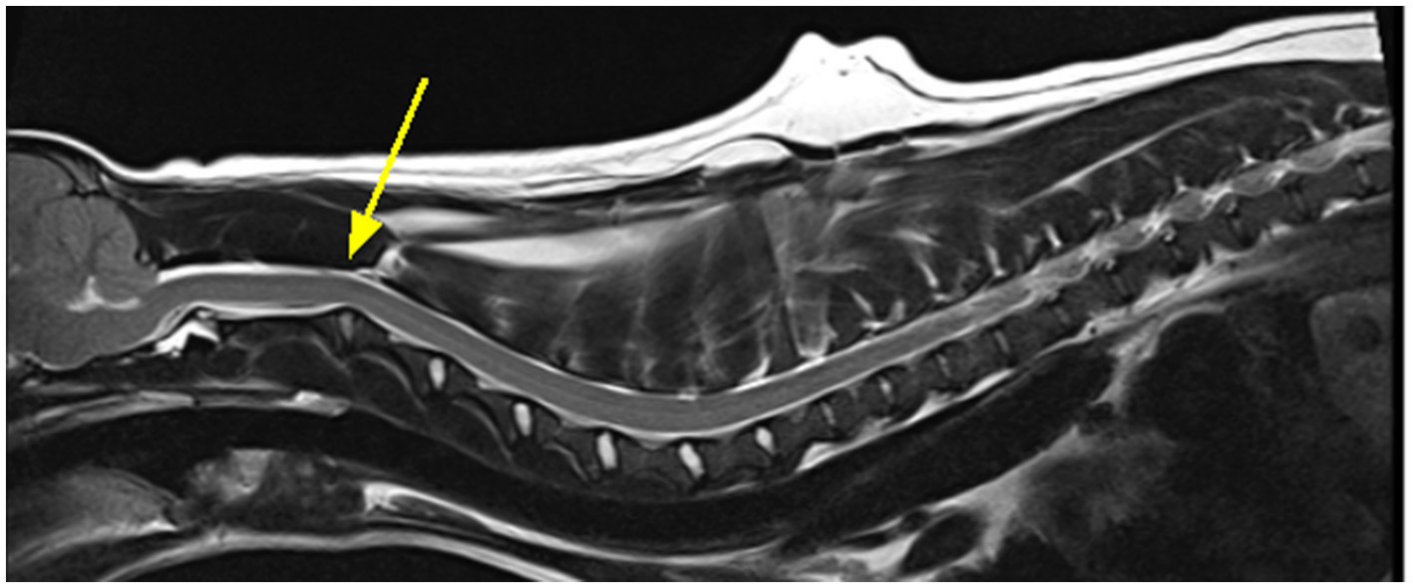
Exemplar MRI scan of a CKCS with CM-P, with a bold yellow arrow pointing to a site of significant curvature at the C2/C3, as mentioned by Cirovic et al. (24).

In the CKCS spinal cord segment C3 is the area most likely affected by a syrinx however this cannot be the reason that this region was highlighted as significant for CM-P as these dogs do not have syringomyelia. The C2/C3 region is also noted to have a curvature (24), as exemplified in Figure 11. It is possible that this curvature is more pronounced in CM-P affected dogs, but this hypothesis would need to be tested in a further study.

### 4.2 SM-S morphologies and clinical relevance

Like CM-P there were two morphologies outside the central nervous systems and being very close together (soft palate) or overlapping (larynx) with the CM-P biomarker morphologies. Since SM is secondary to CM this strengthens the argument that, however surprising, these areas may be significant for the disorder either as para—phenomenon or disease pathogenesis for example affecting Valsalva maneuver mimics.

The region of the rostral cerebellum is more easily explained, as in CM this region is typically displaced caudally and deformed by the occipital lobe which is displaced caudally by the craniofacial hypoplasia and rostral neuroparenchymal overcrowding (2). In SM the tentorium cerebelli is typically reduced or more horizontal (4). Likewise, the brainstem is typically deformed and displaced caudally and is often “kinked” immediately caudal to the region of interest. The region of interest is directly ventral to the 4th ventricle which is typically dilated in SM-S and so it is possible the morphological changes reflect these phenomena.

There is significant confidence in these finding given the strong performance during the initial training and testing, as well as the bootstrapped CM-P testing, giving a much larger dataset to understand how the information learned can generalize across the wider population. The fifty-one dogs in the CM-P dataset are classified with a specificity of 76%, approaching the initial specificity of 80%, indicating the machine is classifying effectively.

### 4.3 Limitations of study

The support vector-based machine learning framework (20) was used due to being well-suited for comparatively smaller datasets through use of techniques derived from linear algebra, as compared to other deep learning-based approaches which require an order of magnitude larger dataset (which was unavailable for this work). An initial rigid-body registration was needed for approximate alignment prior to Demons (non-linear image) registration; this may potentially obscure the morphologies of larger biological structures. Additionally, the registration process depended on a reference dog that is representative of the dataset at large; however, the reference dog used may, or may not, be a true representation across a much larger population. Therefore, to increase ecological validity, further data would be needed in the future, and this may reveal a reference subject subtly different to that used here.

In this study the imaging was obtained by the same team of experienced qualified radiographers, using a single high field MRI machine and with optimal positioning of the dog. However, much imaging in veterinary medicine is not performed to such a high standard and is limited by suboptimal positioning, low field MRI and obtained by operators with no MRI specialist qualifications and minimal training. Any machine learning technology solutions for analysis of large datasets will need to overcome these challenges. In addition, the current CMSM health scheme (25) does not require imaging that includes the entire brain which limits the use of historical images, and such dogs would have to be accurately phenotyped.

### 4.4 Future work

Future research would benefit from a larger dataset for enhanced generalizability of the ML predictions which could result in an application that could be used for pre-breeding health screening and as a tool for diagnosis of CM-P. However, such a tool would have to be adaptable to variations in imaging quality. CM-SM is a consequence of a three-dimensional change in morphology however assessment is currently made from a single two-dimensional midsagittal image. This failure to account for three-dimensional changes is a major limitation and so the next steps in the Canine Chiari Group Head Space Project will be to extend this approach to three-dimensional morphologies.

Although this work was focused on CM-P and SM, the same methodology might also be developed for application to other brachycephalic breeds and prediction of other associated conditions such as Brachycephalic Obstructive Airways Syndrome (BOAS).

## 5 Conclusions

The results suggest the proposed methodological pipeline could successfully analyze morphological changes experienced as a result of developmental disorders, in a way that was entirely machine-led and presumed no prior biological knowledge, using an entirely data-driven approach and attaining strong performance during the training and testing. The internal morphologies of the CKCS, subject to CM-P and SM-S, are now better understood not just with neuroanatomical regions of interest, but also the behaviors associated to their respective deformations. This study also allowed the distinction of morphologies that are indicative of normal neuroanatomical deformations, which are not linked to disease. Although these may be para-phenomenon it may be useful as a means to screen for CM-P/SM-S tendency.

This work serves as a proof of concept for the morphological examination of diagnostic medical imaging in the detection of CM-P and SM-S. These results represent state of the art performance in using machine learning architecture as a diagnostic tool for morphological assessment of neuroanatomical disorders.

## Data Availability

The raw data supporting the conclusions of this article will be made available by the authors, without undue reservation.
